# The Adenovirus Vector Platform: Novel Insights into Rational Vector Design and Lessons Learned from the COVID-19 Vaccine

**DOI:** 10.3390/v15010204

**Published:** 2023-01-11

**Authors:** Erwan Sallard, Wenli Zhang, Malik Aydin, Katrin Schröer, Anja Ehrhardt

**Affiliations:** 1 Virology and Microbiology, Center for Biomedical Education and Research (ZBAF), School of Medicine, Faculty of Health, Witten/Herdecke University, 58453 Witten, Germany; 2 Laboratory of Experimental Pediatric Pneumology and Allergology, Center for Biomedical Education and Research, School of Life Sciences (ZBAF), Faculty of Health, Witten/Herdecke University, 58455 Witten, Germany

**Keywords:** adenovirus, SARS-CoV2, adenoviral vector, vaccine, side effect

## Abstract

The adenovirus vector platform remains one of the most efficient toolboxes for generation of transfer vehicles used in gene therapy and virotherapy to treat tumors, as well as vaccines to protect from infectious diseases. The adenovirus genome and capsids can be modified using highly efficient techniques, and vectors can be produced at high titers, which facilitates their rapid adaptation to current needs and disease applications. Over recent years, the adenovirus vector platform has been in the center of attention for vaccine development against the ongoing coronavirus SARS-CoV-2/COVID-19 pandemic. The worldwide deployment of these vaccines has greatly deepened the knowledge on virus-host interactions and highlighted the need to further improve the effectiveness and safety not only of adenovirus-based vaccines but also of gene therapy and oncolytic virotherapy vectors. Based on the current evidence, we discuss here how adenoviral vectors can be further improved by intelligent molecular design. This review covers the full spectrum of state-of-the-art strategies to avoid vector-induced side effects ranging from the vectorization of non-canonical adenovirus types to novel genome engineering techniques.

## 1. Adenoviruses and Their Vectorization

### 1.1. Features of Adenoviruses

Adenoviruses (Ad) comprise a large natural diversity of >200 identified non-human adenovirus types and >100 identified human adenovirus types [1] (Table 1) which contribute to a variety of clinical conditions, such as respiratory, gastrointestinal, genitourinary, and ocular infections. Human Ad are subdivided into seven different species (A to G) of which species D comprises the largest group, including more than 70% of the identified human Ad types.

After the discovery of Ad as etiologic agents in human adenoids in 1953 [2], historically most basic virology studies were performed with species C viruses represented by human Ad type 5 (Ad5) and human Ad type 2 (Ad2). In 1962 it was demonstrated that human Ad type 12 (Ad12) can cause tumors in newborn hamsters [3]. However, there is no evidence that Ad is associated with tumor formation in humans, and screens for identifying Ad DNA in human tumors have failed, but it is hypothesized that a “hit-and-run” transformation may exist for Ad [4]. This hypothesis claims that cellular transformation is caused by an original “hit” and while maintaining the transformed state the viral molecules are lost (“run”).

**Figure 1:**
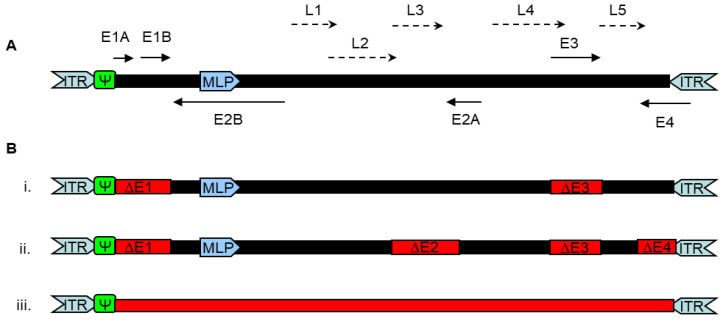
Schematic overview over the wild-type adenovirus and recombinant adenoviral vector genomes. (**A**) Map of the wild-type adenovirus genome and its transcription units. The central, solid line represents (black) the viral genome. Positions of the left and right inverted terminal repeats (ITRs, grey), the packaging signal (Ψ, green), the early transcription units (E1A, E1B, E2A, E2B, E3, and E4 in solid arrow), and the late transcription units (major late promoter (MLP), blue; L1–L5 in dashed arrow) are shown. Arrows indicate the direction of transcription. (**B**) Different generations of recombinant adenoviral vectors used in gene therapy. Elements shown in red represent deletions providing space for insertion of transgene cassette. i. First-generation adenoviral vectors lacking E1 and/or E3. ii. Second-generation adenoviral vectors with multi-deletions. iii. High-capacity adenoviral vectors (HCAdV) with all viral coding regions deleted.

The Ad virus particle is icosahedral-shaped and measures 70 to 100 nm in diameter in a type-dependent manner. The protein shell is composed of 252 capsomeres consisting of 240 hexons, 12 pentons and 12 fiber proteins with a knob domain. Proteins VI, VIII, IX and IIIa are bridging proteins and stabilize the hexon proteins within the capsid. There are four proteins in the core of the virion, of which proteins V, VII and mu are attached to the DNA, and the terminal protein (TP) is covalently attached to the ends of the 26–48 kb dsDNA linear Ad genome. The Ad genome contains two origins of replication (ORI), namely the inverted terminal repeats (ITR) at both ends. It also carries the four early transcription units E1 to E4 and the five late transcription units L1 to L5 (Figure 1).

Ad enter the host cell utilizing different receptors on the cell surface including the coxsackievirus and adenovirus receptor (CAR), cluster of differentiation 46 (CD46), desmoglein-2 (DSG2), sialic acid (SA) and heparin sulfate proteoglycan (HSPG) (Table 1). After binding to the host cell, predominantly via the fiber protein, the early phase of infection starts with clathrin-mediated endocytosis, followed by Ad DNA translocation to the nucleus and transcription of the early genes. Afterwards, late transcription units including the structural proteins are expressed. The Ad DNA replicates within the nucleus, and encapsidation of the viral DNA into translated capsid proteins is initiated. For Ad5, it was demonstrated that virus particle assembly takes place in the nucleus with a packaging constraint of 105% of the wild-type genome length [5], and, 38 to 48 h after infection, approximately 10,000 to 100,000 progeny viral particles per cell are released. This facilitates high titer rescue and renders the adenovirus system attractive for vector development in virotherapy to treat tumors, and for gene therapy and vaccination studies. Of note, analyses regarding the packaging constraint of other types are lacking and the constraint may therefore differ.

**Table 1:** Overview of the identified adenovirus types [6,7,8]. Summarized are the seven adenovirus species, their general tropism and the receptor type. Vectorized adenovirus types according to PubMed records are underlined.

Species	Human Adenovirus Types	Tissue Tropism	Receptor (Ad Type)
A	12, 18, 31, 61	Intestine	CAR ^b^ (12)
B	3, 7, 16, 21, 50, 66, 89, 11, 14, 34, 35, 55, 68, 89, 106, 76–79	Tonsils and respiratory tract, hematopoietic cells, kidney, urinary bladder	CD46 (3, 7, 11, 14, 16, 21, 34, 35, 50) DSG-2 ^c^ (3, 7, 11, 14, 55) HSPG, CD80/86
C	1, 2, 5 ^a,f^, 6, 57, 104, 108	Respiratory tract Ad5 infect liver in mouse	CAR (1, 2, 5, 6), HSPG ^e^ MHC-I, VCAM-I, integrins
D	8–10, 13, 15, 17, 19, 20, 22, 23, 24, 25, 26 ^a^, 27, 28, 29, 30, 32, 33, 36, 37, 38, 39, 42, 43–47, 48, 49, 51, 53, 54, 56, 58–60, 62–64, 65, 67, 69, 70, 71, 72, 73–75, 80, 81–88, 90–103, 105, 107, 109–113	Eye, conjunctive tissues	CAR (9, 10) SA ^d^ (8, 19a, 37) CD46 (17, 26, 48, 49)
E	4	Respiratory tract, eye	CAR
F	40, 41	Intestine, enterocytes	CAR, HSPG
G	52	Intestine	CAR, SA

^a^ Vector types used in the CARS-CoV pandemic; ^b^ Coxsackievirus and adenovirus receptor, ^c^ DSG2: desmoglein 2, ^d^ SA: sialic acid, ^e^ HSPG: heparan-sulfate proteoglycan; ^f^ most commonly used vector type.

### 1.2. Adenoviral Vectors

Advantages of using Ad vectors (AdV) for delivery include their episomal, and thus non-integrating nature, their large transgene capacity and the option to produce replication-competent and replication-deficient vectors. The predominantly used cell line for production of recombinant Ad for therapeutic approaches are human embryonic kidney (HEK) 293 cells [9], which can be generated by transfecting Ad5 DNA fragments and subsequent integration of the left arm of the Ad5 genome including E1 inside the cell genome. To prevent potential risk of replication-competent adenovirus (RCA) generation during propagation of El-deleted adenoviral vectors, PER.C6 cells [10] stably expressing E1 transcription unit were generated. In PER.C6 cells, the E1 transcription unit is expressed under the control of the human phosphoglycerate kinase (PGK) promoter preventing recombination between Ad sequences in the production cell line and homologous sequences in the vector. Therefore, acquisition of the E1 transcription unit into the vector can be prevented.

There are different generations of AdV (Figure 1). For replication-deficient first generation AdV, the E1 region is completely deleted and, therefore, inserts of up to 5 kb can be placed into the E1 region. To increase the transgene capacity up to 8 kb, the E3 gene region can additionally be deleted. These first generation AdV are frequently used and are mainly based on Ad5, which can be produced in HEK293 or PER.C6 cells. In order to rescue replication-deficient first generation vectors from non-Ad5 types, one option is to stably integrate the cognate E1 transcription unit of the respective Ad type into the producing cell line [11]. As an alternative option, regular HEK293 or PER.C6 cells can be used for production of non-Ad5 vectors if the Ad5 E4 transcription unit is supplemented in cis or in trans. This is because the complexes formed by the proteins E1 55K and E4 open reading frame (ORF) 6 play an essential role in viral replication by selectively transporting viral mRNAs and shut-off the host cell protein synthesis [12]. Therefore, to enable the production of other Ad types in these Ad5 E1 stably expressing cell lines, the E4 ORF6 region in the respective genome of the Ad type of interest needs to be replaced by the corresponding region from Ad5 [13]. Another interesting option would be to test whether the E1 region of one Ad type can substitute and support replication of another Ad type.

Besides deletion of E1 and E3 regions, second generation AdV are deleted for further ORF in the early gene regions (E2 and/or E4), which increases packaging capacity. However, this also renders the vectors more difficult to handle, because amplification rates may be reduced, and lacking ORFs need to be complemented in trans by the producer cell line [14,15]. The newest generation of adenoviral vectors, termed “gutless”, helper-dependent or high-capacity adenoviral vectors (HCAdV), are deleted for all viral coding sequences enabling packaging of foreign DNA of up to 36 kb [16,17,18].

Historically, most of the AdV are based on Ad5, but it has become evident that the natural diversity of all Ad types needs to be explored. To expand the tropism of Ads, engineered chimeric AdVs based on the commonly applied Ad5 containing modified fiber proteins derived from other Ad types (for example, Ad11, -35, -3, -40 and -37) have been introduced [19,20,21,22]. In most cases, the chimeric AdVs demonstrated higher transduction efficiency and more potent tumor cell killing. However, the modification in the Fiber and/or knob region does not overcome immune responses against other components of the virus such as hexon and penton. Thus, the generation of novel AdVs completely based on a different serotype is attractive. Our previous review summarized technologies to get genetic access to alternative adenoviral genomes using methods such as traditional molecular cloning with restriction enzymes, cosmid-based methods with customized-module selection and modification, homologous recombination in eukaryotic cells and homologous recombination in bacteria [23]. Here we provide an overview of the recent techniques which we deem most promising for high-throughput genetic engineering and vectorization of Ad.

**Figure 2:**
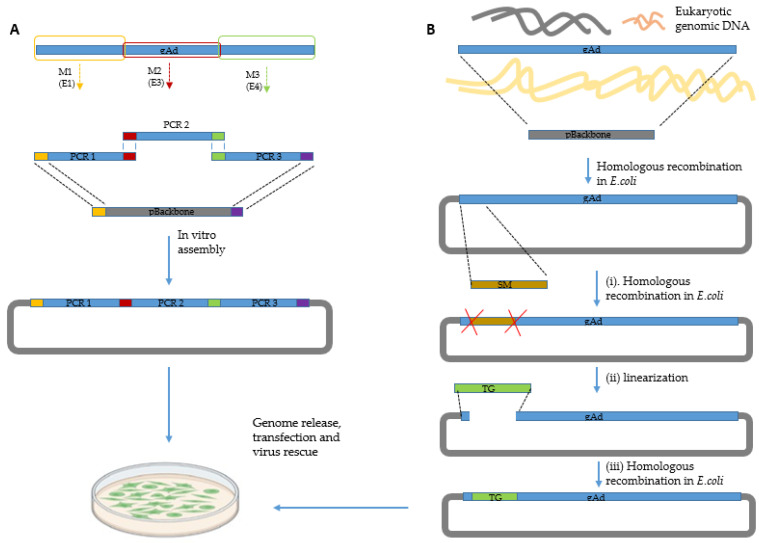
Cloning and rescue of recombinant adenoviral vectors. (**A**) Modular assembly of adenovirus genomes. Modifications need to be introduced into individual modular, before the final assembly. The figure above shows the modification of adenovirus eerily gene region (E1, E2 and E3). (**B**) Direct cloning of complete adenovirus genomes from purified virions or infected cells and further modification of adenovirus genomes by homologous recombination. For the adenovirus genome engineering, ampicillin-mediated positive selection is used insert the selection marker cassette (SM, ccdB-Amp) in the first step (i). Then, the counter-selection marker containing adenovirus plasmids are treated with restriction enzymes to linearize the plasmid (ii). Finally the linearized adenovirus genome containing plasmid, and the insert flanked by homologous arms to the target region of the adenoviral genome are co-electroporated into the E. coli strain GB05-dir, in which the RecET recombinase is induced with L arabinose to express, enabling linear-linear homologous recombination to replace the ccdB-Amp cassette with aimed insert. Afterwards, the recombinant adenoviral vector can be reconstituted by transfection of the linearized plasmid into the producer cell line (such as HEK 293 cells) for further applications. Created partly with Biorender.com.

Recent methods utilize modular assembly of complete viral genomes and advanced recombineering techniques for cloning of complete adenovirus genomes from different sources and further genetic modification (Figure 2). For modular assembly, the inserts, for instance complex transgenes [24] or even the complete adenovirus genomes [25], are ligated using the Gibson assembly technique with isothermal DNA assembly technique to seamlessly fuse DNA fragments with short overlaps [26,27,28,29]. The Gibson Assembly reaction is carried out under isothermal conditions using three enzymatic activities: a 5′ exonuclease generates long overhangs, a polymerase fills in the gaps of the annealed single strand regions, and a DNA ligase seals the nicks of the annealed and filled-in gaps [26,30]. Gibson assembly has already been used to clone the fowl adenovirus 4 genome into a pBR322 plasmid backbone [31]. Moreover, site-directed modification of an adenoviral vector genomes has been achieved by combination of Gibson Assembly and restriction mediated-ligation [28]. However, insertions or gene replacement directly in the adenoviral genome can be performed by Gibson Assembly only if unique restriction sites delimiting the region to be modified are already present, which substantially limits the applicability of this technique. Otherwise, it is necessary to use different techniques or to reassemble the entire AdV genome, including the desired modifications by Gibson Assembly of several overlapping fragments [25]. Furthermore, the fragment size for assembly is limited by around 20 kb.

Based on older developments [32,33,34,35], advanced recombineering techniques have recently been established [36,37,38]. The desired Ad genome can not only be cloned from purified virus preparations but also from virus infected cells [39]. Moreover, this is suitable for the seamless modification of any locus. Most traditional recombination protocols rely on red proteins from a lambda phage to mediate recombination between a circular and a linear fragment. However, poor efficiency for sequences with larger size (>10 kb) or high GC content limits throughput for many applications. Furthermore, it was recently demonstrated that double-strand break generated by CRISPR/Cas9 can highly increase the homologous recombination efficiencies in mammalian genomes [40]. In concert with our observation that homologous recombination from linear fragments promoted by the full-length Rac prophage protein RecET results in substantially higher cloning efficiency, we recently developed a strategy to convert the ccdB-mediated counter selection from linear–circular homologous recombination to linear–linear homologous recombination [38].

Based on these methods and tools for generation of non-Ad5 vectors, single Ad types were converted into vectors for therapeutic approaches and vaccination studies. For vaccination, for instance, Ad26 was broadly explored in the COVID-19 pandemic which is discussed in more detail in the next section. Mainly Ad5, but also non-Ad5 adenovirus-based vectors have been explored in cancer treatment. Among prominent examples are Ad3-based oncolytic viruses which were analyzed in tumor patients. In one study an Ad3-based vector in which the endogenous E1A promoter was replaced by a human telomerase (hTERT) promoter resulted in tumor killing in both animal models and tumor patients [30]. Switching the Ad type may also enable re-administration of an oncolytic Ad and repeated dosing in humans, because neutralization after administration of a commonly used Ad5-based vector can be circumvented. In particular, in such patients with treatment resistances against traditional therapy, Ad-based gene delivery represents a promising alternative for successful cancer treatment. This has already been reviewed elsewhere [41,42,43,44,45] and is not the focus of this article.

## 2. Adenoviral Vectors Used in the COVID-19 Pandemic

Besides mRNA-based vaccines, four adenoviral vector platforms have been utilized to combat the pandemic caused by severe acute respiratory syndrome coronavirus 2 (SARS-CoV2): ChAdOx1, Ad.26.COV2.S, Sputnik V and Convidecia. Here we will solely focus on the vector setup. All Ad-based vectors are first generation vectors which are replication-deficient and therefore do not result in productive infection. Regarding safety issues, it is important to emphasize that these vectors enter the cells, express the spike protein, and do not initiate the normal Ad infection cycle. Furthermore, they deliver their genetic payload to the nucleus, but integration events are extremely rare and, in contrast to other vector systems such as adeno-associated virus vectors or retroviral vectors, there has been no report of insertional mutagenesis after transduction with an adenoviral vector.

### 2.1. Adenoviral Vectors Explored in the SARS-CoV2 Pandemic

The Ad vector platform providing the basis for the development of ChAdOx1 was introduced in 2012 [32] and for Ad26.COV2.S in 2007 [13]. ChAdOx1 is based on the chimpanzee Ad isolate Y25, which was amplified in HEK293A cells, a subclone of HEK293 cells. This cell line is characterized by its flat morphology, which is thought to facilitate initial production, amplification, and titration, viral DNA purified to generate a bacterial artificial chromosome-based vector for genetic manipulation of virus genomes and virus rescue. Using galactokinase (galK)-based recombineering [46], further genetic modifications were introduced by deleting the complete E1 and E3 regions and in the E4 region ORF4, ORF6 and ORF6/7 were replaced with Ad5 equivalent virus genome regions. To ensure amplification in these cells, it is essential that complexes between the proteins E1 55K and E4 ORFs can be formed supporting viral replication by selectively transporting viral mRNAs and by shutting off host cell protein synthesis. Besides the application in the SARS-CoV2 pandemic, the ChAdOx1 vaccine vector platform was also involved in other vaccination studies including vaccines against influenza A, Mycobacterium tuberculosis, MERS-CoV and SARS-CoV1, showing that comprehensive data sets to assess this vaccine setup were accumulated before.

Ad26.COV2.S is based on the human Ad type 26 (Ad26) and was generated by homologous recombination in the packaging cell line PER.C6 [13,47]. Here a pAdApt adaptor plasmid containing the left end of the Ad genome with the left ITR, a deletion in the E1 region and the spike protein expression cassette was used for recombination with a pWE cosmid containing the remaining parts of the Ad genome, from the pIX encoding sequence to the right ITR. TheAd26-based vector platform was also explored in other clinical studies including vaccination against human immunodeficiency virus (HIV), ebolavirus, Plasmodium, RSV, HPV and filovirus.

Sputnik V (Gam-COVID-Vac) is based on a heterologous vaccine comprising two AdV based on recombinant first generation Ad5 and Ad26 vectors, which were injected sequentially [48]. In addition, the Ad5-vectored COVID-19 vaccine Convidecia mainly administered in China is based on first-generation Ad5 vector. Here, we focus on Ad26.COV2.S and ChAdOx1, because these two vector types were more broadly applied in the SARS-CoV2 pandemic [49].

The ChAdOx1 and Ad.26.COV2.S vaccines were purposedly constructed from Adenovirus types with very low seroprevalence to avoid a potential decrease in efficiency due to pre-existing immunity. However, the highly seroprevalent Ad5-derived vector used in Sputnik V did not prevent vaccinees to develop strong anti-COVID immunity, and for the same vaccine no correlation was found between pre-boost anti-vector antibody titers and boost efficacy [50]. This finding corroborates the results of unrelated clinical trials on Ad5-based anti-HIV vaccines [51].

### 2.2. Risks and Side Effects of the SARS-CoV2 Vaccine Vectors

Several minor side effects, such as local reaction at the puncture site and systemic effects (e.g., fever, muscle aches), have been observed, and a major side effect represented by vector-induced Thrombotic Thrombocytopenia (VITT). This disorder occurs in around 1 in 100,000 vaccinations and is fatal in 23–40% of all reported cases [52]. Its clinical presentation is reminiscent of heparin-induced Thrombocytopenia (HIT), in which platelet factor 4 (PF4) complexes with heparin and forms neoantigens [53]. The reason why only a tiny minority of patients receiving AdV-based COVID-19 vaccines develop VITT is still unknown. It has been hypothesized that intrinsic factors, either acquired or genetic, can predispose a few subjects to develop VITT [54]. For instance, patients with VITT had high levels of antibodies against PF4, although they were never treated with heparin. Furthermore, it was shown that PF4 can bind to the incoming viral vector [55,56], which may induce anti-PF4 antibodies by forming Ad-PF4 complexes, similar to HIT mechanisms. Indeed, molecular modeling performed by Baker and colleagues suggested that the positively charged PF4 protein binds to negatively charged interhexon spaces [56], which may therefore function like heparin molecules. It was also speculated that vaccine-associated antiplatelet autoantibodies may be generated and cause in vivo platelet and blood clotting activation [57], or that soluble spike protein may damage endothelial cells by binding to angiotensin-converting enzyme 2 (ACE-2) on endothelial cells [57].

After it became known that ChAdOx1 nCov19 and Ad26.COV2.S were associated with an increased rate of thromboembolic events, rapid changes in vaccination strategy took place, indications were reevaluated, and larger multicenter studies followed to determine the exact causes [58,59,60,61]. Neurologic symptoms including headache, focal deficits, or movement disorders have also been reported after SARS-CoV-2 vaccination [62]. Myocarditis following mRNA vaccines have also been described, especially in younger and primarily male adults. Furthermore, a few cases of autoimmune vasculititis have been described following the Pfizer-BioNTech COVID-19 vaccine [63]. To what extent these isolated side effects are caused by the vaccinations will, hopefully, be determined by further longitudinal studies. Features of ChAdOx1 and Ad26.COV2.S vaccine vectors are summarized in Table 2. 

Overall, AdV-based COVID vaccines established the Ad platform as one of the fastest, cheapest and most efficient vaccine types to react to pandemics. New vaccines could be designed, produced at large scale and vetted through the entire process of clinical trials in under one year, and since then have been administered well over 2 billion times worldwide. Nevertheless, the Ad vaccines efficiency was lower than mRNA vaccines [64] and severe safety issues occurred. Further improvement in vector design and construction are warranted to unleash the full potential of the Ad vector platform.

## 3. Strategies to Improve the Adenovirus Vector Platform

### 3.1. Safety Challenges Faced by the Ad Vector Platform

Depending on the application route, administered Ad vaccine, gene therapy or oncolytic virotherapy vectors can be exposed to components contained in the blood stream, the innate and adaptive immune system, and various other cell types. Local administration (e.g., intramuscular or intratumoral) is considered safer than intravenous application, but depending on the target issue is not always feasible, and small amounts of vectors are still exposed to blood components due to leakage into the vasculature, particularly in the case of inflammation [55]. The following paragraph summarizes major safety issues, which have been predominantly studied based on Ad5, the most frequently used Ad vector.

AdV released in the bloodstream can be trapped by liver-specific phagocytic Kupffer cells and macrophages by binding to scavenger receptors [65]. Furthermore, Ad fibers bind to platelets [66], for which several interaction mechanisms have been hypothesized, such as binding to CAR or platelet-specific integrins on the platelet surface. Ad was also shown to bind to human erythrocytes via complement receptor 1 (CR1) and CAR [67]. More generally, vectors with wild-type capsids usually suffer from a broad to generalist tropism, leading to vector sequestration in non-target tissues, and low transduction efficiency in many potential target cells. This can prompt physicians to use unsafely high doses and prompt tissue toxicity.

Other important interaction partners in the blood are extracellular proteins such as the coagulation factor X (FX) that binds to the hypervariable regions (HVR) 5 and 7 loops of the hexon and bridges Ad to HSPGs on the cell surface [68]. Furthermore, immune complexes caused by antibody (IgM, IgG, IgA) binding to Ads stimulate dendritic cell maturation. Besides these interaction partners, the classical and alternative pathways of the complement play an important role in Ad-host interactions [69]. Binding of FX to the virion prevents complement and IgM binding [70]. Careful analyses of binding partners and their affinity may shed more light on these interactions and whether they interfere with virus-receptor mediated uptake.

Finally, vector immunogenicity needs to be carefully modulated depending on the desired application and target population. For example, gene therapy requires vectors with low immunogenicity to facilitate efficient gene delivery and long-lasting expression, while oncolytic virotherapy would, on the contrary, benefit from a localized and controlled anti-vector immune responses within the tumor, which optimally should also prime immune responses against tumor antigens. In the case of vaccines, the immune response should be strong, durable and boostable, but directed primarily against the antigen instead of the vector itself. Furthermore, the age and health status of the target population, as well as the vector seroprevalence, should be taken into consideration during vector choice and potential engineering. The intramuscular injection of COVID-19 vaccines may result in local side effects, e.g., tenderness, pain, and inflammation at the injection site. Systemic complications after vaccination are low, and may be related to Guillain-Barre-Syndrome after Johnson&JohnsonJ/Janssen COVID-19, mycocarditis and pericarditis after Moderna or Pfizer-BioNTech (https://www.cdc.gov/coronavirus/2019-ncov/vaccines/safety/adverse-events.html (accessed on 1 January 2023)) [71].

**Figure 3:**
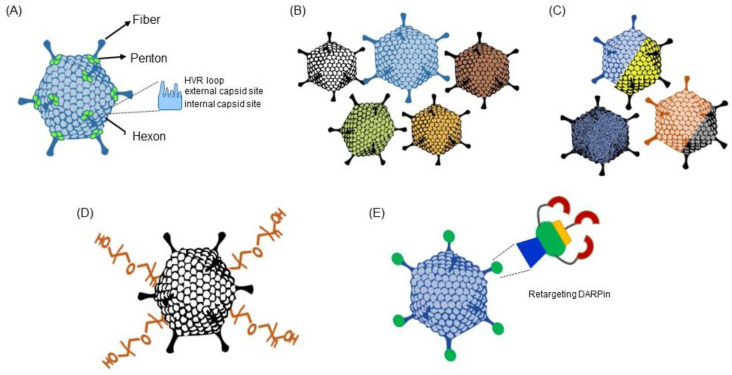
Strategies to improve AdV. (**A**) Genetic modification of viral capsid proteins (hexon, fiber, penton). The protein can either be exchanged by the respective protein of another Ad type or be mutated. (**B**) Adenovirus serotype switch. This strategy is based on vectorization of alternative human and non-human adenovirus types. (**C**) Directed evolution. Features can be enhanced by directed evolution using selection procedures after infection with a library of different Ad variants. (**D**) Chemical capsid modification can be applied to shield the Ad particle from unwanted binding to cells or blood components after systemic administration. (**E**) Bispecific DARPin molecules can be attached to the Ad fiber retargeting the vector to another cell surface structure.

In order to modulate interactions with host proteins, tissues and immune cells, and to improve the outcome of a viral vector therapy or vaccination, several strategies can be pursued, which are outlined in the next section and summarized in Figure 3.

### 3.2. Intelligent Engineering of the Adenovirus Vector Platform

Genetic modification of exposed virion protein domains can be explored, particularly for the hexon, fiber and penton proteins. For instance, generating a chimeric Ad5-based vector by simple exchange of hexon HVR loops protected against pre-existing anti-vector immunity [72]. HVR exchanges and even point mutations were shown to ablate FX binding [73]. Moreover, peptide insertions in exposed loops of the fiber or the hexon facilitated vector retargeting and decreased unwanted interactions with notable versatility [74,75].

The wide diversity in tropism, immunogenicity, seroprevalence, safety profile, transduction time course and replication strength of non-Ad5 virus types, may be harnessed to customize vectors for the desired application. Early studies showed that serotype exchange allows sequential administration of AdV [76]. Since then, methods for cloning a broad variety of different Ad types have been developed, and vector platforms have been established for numerous non-canonical Ad types [23]. The ChAdOx1 and Ad26-based COVID vaccines result from these efforts to vectorize low prevalence Ad types, but the gene therapy and oncolytic virotherapy fields remain dominated by a limited number of types regardless of the desired application.

Since the optimal modifications required to achieve a therapeutic purpose such as vector retargeting are not always known beforehand, improved vectors may be generated by directed evolution as an alternative to rational design [77]. The establishment of directed evolution workflows has long been hampered by the lack of high-throughput Ad variants library construction techniques, but the recent developments in Ad cloning outlined above may help to bridge this gap.

In addition to direct capsid modification, retargeting and immunogenicity modulation may be achieved by decorating virions with designed ankyrin repeat proteins (DARPins) [78] or other types of bispecific adapters, as well as through chemical modifications of the capsid [79]. Chemical shielding was first performed in 1999, showing that PEGylation (covalent addition of polyethylene glycol polymers at chosen sites of the capsid) protects from attacking neutralizing antibodies [80]. Recently, Ad COVID candidate vaccines with peptide-based capsid shielding were able to induce more than a tenfold higher neutralizing antibody titers against SARS-CoV-2 than non-shielded counterparts due to a lower anti-vector immunity, and are now investigated in clinical trials [81]. Therefore, the different approaches of capsid chemical engineering can improve the safety profile and efficiency of vectors, and may achieve even greater effects if used in combination. However, a limitation of post-translational capsid modifications is that they introduce further steps in the manufacture and increase the cost, making them less attractive approaches for large-scale applications such as vaccination campaigns.

A critical challenge facing Ad vaccines is epitope masking, a phenomenon whereby the anti-vector immune response restricts in breadth and intensity the response directed against the desired antigen [82,83]. This process may play a role in the comparatively low efficiency and low robustness against SARS-CoV-2 variants of Ad COVID vaccines [64], given that no measure has been taken to counter it apart from the use of replication-deficient vectors. Capsid shielding, serotype exchange and the use of HCAdV may represent efficient steps in this regard [84]. HCAdV display additional advantages as vaccine vectors due to their very large packaging capacity because besides the antigen, cytokines can be expressed to improve and direct the immune response. The molecular design of the antigen-expressing transgenes could be optimized, or several antigens could be expressed from a single vector.

### 3.3. Avoiding PF4 Binding and Subsequent Platelet Activation to Avoid VITT

**Figure 4:**
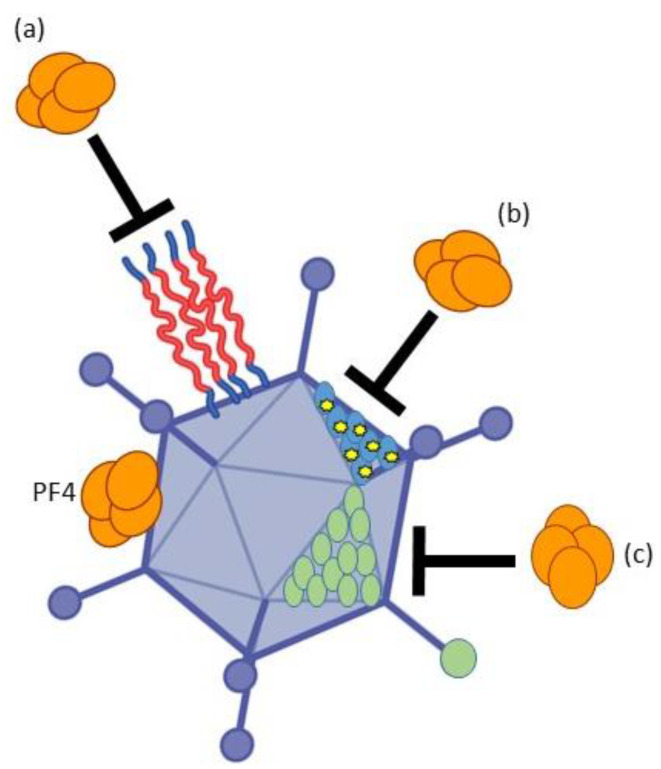
Strategies to avoid binding of blood proteins to the adenovirus capsid. (**a**) Chemical shielding. Using polymers linked to the adenovirus, hexon protein can avoid binding of blood proteins. (**b**) Genetic mutation of the adenovirus hexon protein coding sequence within the adenovirus genome. (**c**) Complete serotype switch using an alternative adenovirus type not binding blood components. In part created with BioRender.

PF4 binds to the hexon of adenovirus vaccine virions [85]. Deletion or chemical shielding of the hexon HVR1 loop was sufficient to ablate detectable PF4 binding of Ad5, which already points towards two methods to design safer AdV with decreased or suppressed risk of VITT (Figure 4). Narrowing down the exact binding site of PF4 to the hexon protein may provide additional options using genetic or chemical engineering of the PF4 binding site. Alternatively, human or non-human AdV types devoid of PF4 binding could be identified, such as Ad34 according to preliminary findings.

### 3.4. Ad Vaccine Vectors Manufacturing and Administration Optimization

Besides these engineering options, better purified preparations without EDTA and human proteins, which are inflammatory and a major cause of side effects with the ChAdox1 vaccines [55], should be made mandatory for clinical use. Moreover, the vaccine administration dose may be optimized with the help of computer models [86] or dose fractionation, which consists of using a lower dose for the prime than for the boost and was associated with higher quality of the immune response [87]. Besides these factors, the administration route may be imporant. For instance, nasal vaccines may be superior to intramuscularly applied vaccines to protect against respiratory infections. Indeed, aerosolized vaccines can, in theory, prime mucosal immunity more efficiently, decrease the risk of side effects, and alleviate logistic issues by requiring lower doses and being less sensitive to cold chain disruptions [88]. The Ad5-based Convidecia vaccine displayed similar efficiency and safety after intranasal administration than after intramuscular injection in a phase 1 clinical trial [89]. A worldwide phase 3 trial is currently ongoing and should give highly valuable information to guide the choice of administration route.

## 4. Conclusions

The natural diversity of Ad, and the option to engineer Ad vectors, provides a great opportunity for further optimization of Ad vectors to avoid side effects, improve manufacturability and augment therapeutic effectiveness. The engineering methods outlined in this article, most notably serotype exchange, capsid genetic and chemical engineering, or the use of HCAdVs, have the potential to tackle all the main safety challenges currently faced by Ad vectors. The vaccination campaign shed further light on the performance of Ad vectors, which, thanks to rapid design and very large-scale production, have stood at the forefront of pandemic containment. As an extremely rare side effect, VITT was probably discovered only because of the unprecedented number of individuals who were treated with a viral vector as part of the worldwide vaccination campaign against COVID-19. The information gained will be extremely useful for future generations of Ad vectors with an improved safety profile and enhanced efficacy for all types of clinical applications.

## Figures and Tables

**Table 2:** Features of ChAdOx1 and Ad26.COV2.S as vaccine vectors in the COVID-19 pandemic. Summarized are the molecular features, clinical studies, risks and side effects.

Vector Type	Chimpanzee, ChAdY25 ChAdOx1 nCoV-19 (AZD1222)	Human Ad ^b^ Type 26 Ad26.COV2.S
Molecular setup	Deletion of E1 and E3 E4 ORF4, ORF6 and ORF6/7 replaced with Ad5 equivalent	E1 Deletion (bp 463-3364) E3 Deletion (bp 26,690-30,682) E4ORF6 replaced with Ad5 equivalent
Clinical vaccination studies	Influenza A, Mycobacterium tuberculosis, MERS ^c^ -CoV, SARS-CoV1	HIV ^d^, Ebolavirus, Plasmodium, RSV ^e^, HPV ^f^, Filovirus
Risks and side effects	Local reaction at puncture site, systemic (e.g., fever, muscle aches), VITT ^a^	Local reaction at puncture site, systemic (e.g., fever, muscle aches), VITT ^a^
Dose and route of administration	5 × 10^10^ vector particles intramuscular	5 × 10^10^ vector particles intramuscular
Administration schedule	Two doses at 4 to 12 weeks of interval	One dose

^a^ VITT: Vector-induced Thrombotic Thrombocytopenia, ^b^ Ad: adenovirus, ^c^ MERS: Middle East Respiratory Syndrome, ^d^ human immunodeficiency virus, ^e^ RSV: respiratory syncytial virus, ^f^ HPV: human papillomavirus, ORF: open reading frame, E1: early adenovirus gene region 1, E3, early adenovirus gene region E3, E4: early adenovirus gene region E4.

## Data Availability

Not applicable.
